# Role of serum matrix metalloproteinase in the diagnosis of gastric cancer

**DOI:** 10.12669/pjms.36.5.2059

**Published:** 2020

**Authors:** Jian Liu, Liya Zhou, Sanren Lin, Bei Yao

**Affiliations:** 1 Jian Liu, Department of Gastroenterology, Peking University Third Hospital, Beijing, 100191, China; 2 Liya Zhou, Department of Gastroenterology, Peking University Third Hospital, Beijing, 100191, China; 3 Sanren Lin, Department of Gastroenterology, Peking University Third Hospital, Beijing, 100191, China; 4 Bei Yao, Department of Laboratory Medicine, Peking University Third Hospital, Beijing, 100191, China

**Keywords:** Gastric cancer, Matrix metalloproteinase, Antibody array

## Abstract

**Objective:**

To determine the clinical value of a matrix metalloproteinase (MMP) antibody array in diagnosing gastric cancer (GC).

**Methods:**

In this prospective study, serum samples of patients with GC (n=66) and non-neoplastic gastric disease (NGD; n=34) were collected between November 2017 and July 2018. The quantitative measurement of 10 MMP-related proteins was done using MMP arrays and compared between the two groups.

**Results:**

The serum levels of MMPs 3, 8, 9 and tissue inhibitor of metalloproteinases (TIMPs) 1 and 2 were significantly higher in the GC group than in the NGD group (*p*<0.05). The area under curve (AUC) of the 10 MMP proteins for the diagnosis of GC varied between 0.500 and 0.658. The total AUC of all MMPs was 0.897 (95% CI: 0.837-0.957). The total AUC of the five MMPs (MMPs 3, 8, 9, and TIMPs 1 and 2) was 0.821 (95% CI: 0.733-0.909) for diagnosing GC. Also, the 10-factor and 5-factor predictive models had good diagnostic ability for early GC with an AUC of 0.865 (95% *CI*: 0.753-0.977) and 0.749 (95% *CI*: 0.600-0.898), respectively.

**Conclusions:**

The detection of multiple serum MMPs with protein biochip technology is promising to be used as a novel non-invasive tool for facilitating early diagnosis or screening of GC.

## INTRODUCTION

Gastric cancer (GC) is a public health burden in high-risk populations of Eastern Asia.^1^ Early diagnosis and timely treatment are the most effective methods to improve the prognosis of GC. However, most of the patients with early gastric cancer (EGC) are asymptomatic, which make it difficult to identify and diagnose them in a timely manner. Currently, the main strategies used for forming a clinical diagnosis for GC include endoscopic biopsy, imaging studies, and blood tests.^2^,^3^ An endoscopic mucosal biopsy is the gold standard for the diagnosis of GC. However, it is difficult to perform an endoscopy in every suspected patient in China, since an endoscopic mucosal biopsy is invasive, time consuming, and costly. Imaging studies, such as an upper digestive tract radiography and computed tomography (CT), can help in diagnosing GC. But both these studies require special equipments with well-trained operators, making it impracticable for widespread application. Finally, the standard tumor markers, such as carcinoembryonic antigen (CEA), carbohydrate antigen 19-9 (CA 19-9), and CA 72-4, have shown little benefit as a method for screening.^4^ Therefore, there is a need for highly sensitive and specific biomarkers with efficient detection techniques to allow widespread screening of patients for early detection of GC.

GC is a highly invasive tumor with early metastatic potential.^5^ Degradation of the extracellular matrix (ECM) and basement membrane (BM) barriers are the crucial steps in the development of metastasis. Matrix metalloproteinases (MMPs) are a family of zinc-dependent endopeptidases that cause breakdown of ECM and BM and promote neoangiogenesis.^6^ More than 28 distinct MMPs have been identified to date. They are categorized in five subgroups based on their substrate specificity as collagenases (MMP 1, 8 and 13), matrilysins (MMP 7), gelatinases (MMP 2 and 9), stromelysins (MMP 3, 10, and 11), and membrane-type (MMP 14 to 17, 24 and 25). The majority of MMPs are activated outside the cell by other activated MMPs or serine proteinases. However, the endogenous tissue inhibitors (TIMPs) inhibit the active forms of MMPs and regulate the activation processes. The reported studies have found that the imbalance between MMPs and TIMPs lead to tumor cell migration and invasion.^7^ It has been suggested that MMPs may be useful for the early diagnosis, treatment, and prognostication of GC. Previous studies mainly focused on some specific MMPs/TIMPs; however, the entire expression profiling in GC patients remains poorly understood.^8^,^9^ In this case control study, we determined the concentrations of 10 representative MMPs/TIMPs in patients with GC and non-neoplastic gastric diseases (NGD) simultaneously and explored the changes of serum MMPs/TIMPs levels to understand their clinical significance.

## METHODS

### Study design and ethical considerations

The study protocol was approved by the Medical Ethics Committee of Peking University Third Hospital (Approval number: IRB00006761-2016058; Date: July 19, 2017). Informed consent was obtained from each patient. In this prospective study, serum samples were collected from consecutive patients who underwent gastroscopy at our institution from November 2017 to July 2018. The demographic and clinicopathological data of the enrolled patients were collected.

The inclusion criteria were as follows: (1) patients with benign and malignant gastric diseases as diagnosed by mucosal biopsy and/or postoperative pathology; (2) greater than 18 years of age; (3) and patients who had not received any chemotherapy, radiotherapy, or other cancer treatment.

The exclusion criteria were as follows: (1) patients with non-adenocarcinoma gastric tumors such as neuroendocrine carcinoma or lymphoma; (2) gastrointestinal submucosal tumor or gastric polyp; (3) multiple chronic disorders such as chronic renal insufficiency, autoimmune diseases, or concurrent neoplasms on clinical examination, endoscopy, and imaging studies; (4) and pregnant or lactating females.

The diagnostic criteria were as follows: GC was confirmed and classified according to the American Joint Committee on Cancer 2010 guidelines. The diagnosis of gastric dysplasia and chronic gastritis was according to the Padova International Classification and the updated Sydney system, respectively.

### Sample collection, preservation, preparation and detection

Peripheral blood (3-4 ml) was taken from the included patients after overnight fasting. The samples were centrifuged at 3000 rpm at 4°C for 10 minutes. The supernatant was then separated and preserved in 2 ml aliquots at -80°C for subsequent analysis.

For the MMP profiling analysis, Quantibody human MMP arrays (QAH-MMP-1, RayBiotech, USA) were used for quantitative examination of 10 secretory MMPs/TIMPs, namely, MMP-1, -2, -3, -8, -9, -10, and -13 and TIMP-1, -2, and -4, respectively. The protein array slides were spotted with specific capture antibodies. 2× diluted serum samples were added, washed, and incubated with a cocktail of biotinylated antibodies as per the manufacturer’s protocol. The cytokine-antibody-biotin complex were visualized by adding the streptavidin-conjugated Cy3 equivalent dye using InnoScan 300 Microarray Scanner (Innopsys, France). The concentrations of MMPs in samples were determined by comparing signals from unknown samples to the standard curve plotted with standard controls assayed in each array simultaneously.

For *Helicobacter pylori* infection detection, the *H. pylori* infection was determined by Warthin-Starry (WS) staining in all specimens using a *H. pylori* detection kit (Beijing ShiJi HeLi Biotechnology Co., Ltd, Beijing, China). The background tissue was stained with yellow and the nucleus was dyed with brown. If there were a *H. pylori* infection, the bacteria would be stained with black.

### Statistical Analysis

All statistical analyses were performed with SPSS version 20.0 (SPSS, Chicago, IL, USA). Continuous variables conforming to a normal distribution were described as the mean plus or minus the standard deviation (mean ± SD) and compared by an independent *t*-test. Data not conforming to a normal distribution is reported as a median (interquartile range; median [IQR]) and analyzed with a Mann-Whitney *U* test. Statistical significance was defined as a two-sided *P-value* less than 0.05. The predictive efficacy of each MMP/TIMP and their combination for diagnosing GC was conducted with a Receiver Operating Curve (ROC) analysis and assessed by the area under the curve (AUC) and its 95% confidence interval (95% *CI*). The point corresponding to maximum sensitivity and specificity was considered to be the optimal cutoff value. Subsequently, the significant differentially expressed MMPs were cross-referenced by bioinformatics (http://www.expasy.org/vg/index/protein). Gene Ontology (GO) and Kyoto Encyclopedia of Genes and Genomes (KEGG) enrichment analysis were further carried out, intending to search the functions and pathways of the candidate MMPs associated with the occurrence and development of GC.

## RESULTS

### Baseline characteristics

A total of 100 patients were enrolled in this study. Based on a pathological diagnosis, they were divided into a GC group (n=66) and a NGD group (n=34). The GC group included 17 cases of early GC and 49 cases of advanced GC. Among them, 32 cases had well or moderately differentiated tumors, and 34 cases had poorly differentiated tumors. The NGD group included 12 cases of chronic superficial gastritis, 12 cases of chronic atrophic gastritis and/or intestinal metastasis, and 10 cases of gastric dysplasia. No significant difference was observed in age, but significant differences in the male/female ratio and *H. pylori* infection rate existed between the GC and NGD groups (Table-I).

**Table-I:** Clinicopathological Features.

Characteristic	Type	GC (n = 66)	NGD (n = 34)	P-value ^a^
Age (year)^c^		64.8±11.1	59.2±17.1	0.093
Gender (n)	Male	52	14	<0.001^b^
	Female	14	20	
	Positive	21	0	<0.001^b^
*H. pylori* infection (n)	Negative	11	34	
	Missed	34	0	

a P value calculated by t-test or chi square;

b statistical difference; p<0.05;

c Mean ± SD.

### Different MMP profiles between the GC and NGD groups

MMP 9, TIMP 1 and TIMP 2 had high expression (>10,000 pg/ml); MMPs 1 and 3 and TIMP 4 had medium expression (1,000-10,000 pg/ml); and MMPs 2, 8, 10 and 13 had low expression (<1,000 pg/ml; Table-II).

**Table-II:** MMP Levels (pg/ml) in the GC and NGD Groups.

MMP Type	GC Group	NGD Group	P-value ^a^
MMP-1^d^	3312.55(6323.19)	2172.79(5278.32)	0.190
MMP-2^d^	24.08(246.06)	52.65(504.37)	0.411
MMP-3^d^	6974.05(7010.98)	4588.08(5431.57)	0.017^b^
MMP-8^d^	374.39(356.51)	168.60(187.15)	<0.001^b^
MMP-9^c^	47946.64±21434.15	32322.08±18980.58	<0.001^b^
MMP-10^d^	23.30(24.67)	37.92(43.46)	0.007^b^
MMP-13^d^	0(1.32)	0(3.64)	0.681
TIMP-1^c^	42509.41±15062.17	34162.33±11623.16	0.006^b^
TIMP-2^c^	17770.82±6146.85	13894.91±4254.42	<0.001^b^
TIMP-4^c^	1538.58±716.23	1261.51±664.03	0.063

a P value calculated by t-test or Mann-Whitney U-test;

b statistical difference; p<0.05;

c Mean ± SD;

d Median (IQR).

**Fig.1:**
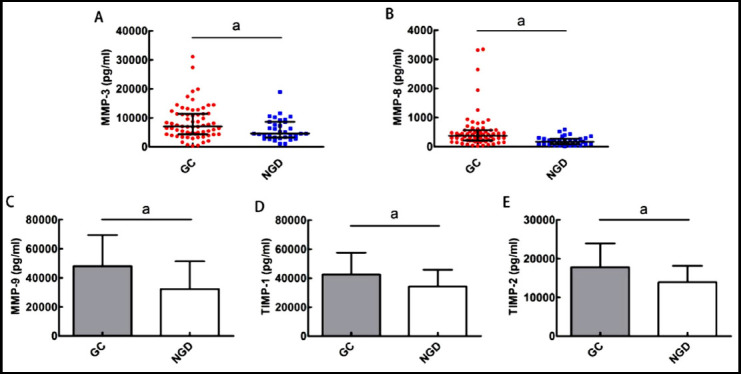
Scatter plots of the concentration distribution of MMP-3 and MMP-8 between the GC and NGD groups (A-B); Histograms of the concentration distribution of MMP-9, TIMP-1, and TIMP-2 between the GC and NGD groups (C-E).

Compared with the NGD group, all the MMP levels, except MMPs 2, 10, and 13, showed an incremental trend in the GC group. Particularly, MMPs 3, 8, 9, 10, and TIMPs 1 and 2 were significantly elevated in the GC group (*p*<0.05). As the concentration of MMP 10 was too low (a median concentration of less than 100 pg/ml), it was eliminated from the screening process. Only the remaining five were retained as differentially expressed MMPs (Fig.1).

### Individual diagnostic value analysis

MMP-2 had the highest sensitivity (83.3%) in the diagnosis of GC, but its specificity was only 41.2%. MMP-3 had the highest specificity (94.1%) but the lowest sensitivity (33.3%). The Youden index (sensitivity + specificity -1) of MMP-8 was the highest, which was 0.430. Its sensitivity and specificity were 63.6% and 79.4%, respectively. Additionally, the AUC of the MMP subtypes for the GC diagnosis ranged from 0.500 to 0.658. Among them, MMP-3 had the highest AUC of 0.658 (95% *CI*: 0.536-0.756; Table-III).

**Table-III:** ROC Curve Analysis for MMPs/TIMPs.

MMP Type	ROC Parameters				

	AUC	95% CI	Sensitivity (%)	Specificity (%)	Cut-off value (pg/ml)
MMP-1	0.580	0.462-0.699	57.6	58.8	2689.84
MMP-2	0.547	0.420-0.675	83.3	41.2	322.21
MMP-3	0.658	0.536-0.756	33.3	94.1	10674.82
MMP-8	0.608	0.671-0.860	63.6	79.4	295.12
MMP-9	0.633	0.608-0.829	72.7	67.6	33617.81
MMP-10	0.583	0.549-0.783	66.7	73.5	28.96
MMP-13	0.500	0.415-0.657	78.8	35.3	2.25
TIMP-1	0.592	0.578-0.787	63.6	73.5	41275.02
TIMP-2	0.608	0.606-0.805	53.0	85.3	18636.72
TIMP-4	0.615	0.500-0.729	48.5	76.5	1602.60

### Combined diagnostic value analysis

**Fig.2:**
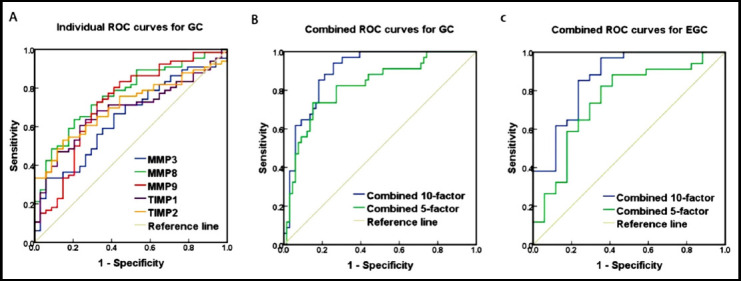
ROC curves for the detection of GC by five individual MMPs and TIMPs (A); ROC curves for the detection of GC or EGC combined with 10 MMPs and TIMPs and five differential MMP and TIMPs (B-C).

The AUC of all 10 MMP/TIMP subtypes in combination for the GC diagnosis was 0.897 (95% *CI*: 0.837-0.957). When 22.142 was selected as the optimal threshold, the corresponding sensitivity and specificity was 74.2% and 94.1%, respectively. Meanwhile, the AUC for the GC diagnosis using the combination of five differentially expressed MMPs and TIMPs was 0.821 (95% *CI*: 0.733-0.909). When 18.488 was selected as the optimal threshold, the corresponding sensitivity and specificity was 73.5% and 84.8%, respectively. Additionally, the 10-factor and 5-factor predictive models showed good diagnostic ability for EGC, with AUC values of 0.865 (95% *CI*: 0.753-0.977) and 0.749 (95% *CI*: 0.600-0.898), respectively (Fig.2).

### Pathway analysis

**Fig.3:**
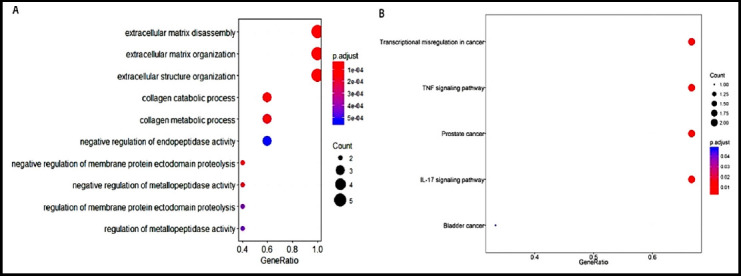
GO biological process enrichment analysis (A) and KEGG pathway analysis bubble plots (B) of five differential MMPs/TIMPs for group GC vs. group NGD.

GO enrichment analysis showed that the differentially expressed MMPs were mostly associated with the processes of extracellular matrix disassembly, extracellular matrix organization and extracellular structure organization. While, KEGG enrichment analysis indicated that they were mainly involved in the process of transcriptional dysregulation in cancer, TNF and IL-17 signaling pathways (Fig.3).

## DISCUSSION

Early detection of GC is important to achieve cure and improve the prognosis of patients with GC. However, because of the heterogeneity of GC tissues, the diagnostic significance of single tumor markers for GC is limited. At present, multiple biological markers are used in combination to improve their clinical utility. Protein and antibody arrays allow us to study the protein expression and protein function in a simple, flexible and rapid manner.^10^ These arrays are used to measure the expression of various proteins from the blood samples of cancer patients and reveal their utility in clinical diagnosis, prognostication, and treatment. For example, Hou et al. detected 12 common tumor markers using a C12 biochip system from the sera of 156 GC patients.^11^ The major tumor markers were CA 19-9 (20.5%), CA 242 (19.9%), CEA (17.3%), and CA 125 (7.1%). However, the detection rate of all four tumor markers in combination was still fairly low in this study (32.7%). It was obviously not enough to screen GC among the high-risk populations. Therefore, new GC-related biomarkers are urgently needed for the enhancement of diagnostic rates.

In recent years, various groups of proteolytic enzymes involved in ECM degradation have been identified, but the MMP group of enzymes are central to the development of tumor invasion and metastasis.^12^ Therefore, they have been useful as diagnostic, prognostic, and predictive biomarkers in several types of tumors, such as esophageal, colorectal, pancreatic as well as GC. Originally, Puig-Costa et al. detected 7 MMPs and 3 TIMPs concurrently in 10 matched pairs of tumor/normal gastric tissues using a MMP antibody array, similar to the present study and constructed a GC-associated ‘MMP/TIMP signature’. Particularly, MMP-8 and -9 and TIMP-1 were found to be significantly upregulated in 90%, 80%, and 70% of GC patients, respectively.^13^ Subsequently, Puig-Costa et al. identified 21 GC-associated inflammatory proteins (including MMP-8 and -9 and TIMP-1) in another cohort of matched pairs (n=10) using antibody microarray-based arrays. They accurately discriminated GC with a sensitivity of 82% and a specificity of 73%. Moreover, MMP/TIMP-associated signaling (cellular morphology, cellular development, and embryonic development) is one of the three major signaling networks associated with the 21-protein INPROGAS (Inflammatory PROtein-driven Gastric cancer Signature) according to an ingenuity pathway analysis.^14^ Similarly, Quan et al. described a 39-protein biomarker assemblage (including MMP-8 and -9) from 507 cytokines to discriminate GC from non-GC tissues (n=8) using normalized array measurements.^15^ Besides, the most enriched of these 39 genes in the KEGG analysis included the TNF signaling pathway similar to the results of our study. These three studies illustrate that there is an inseparable relationship between tumors and inflammation. Detection of various inflammatory factors, especially the MMP family, will help to find novel biomarkers for GC and further explore its pathogenesis and develop new therapeutic targets.

However, all the above studies were based on tissue specimens and had small sample sizes. Tissue specimens can be obtained only by endoscopic biopsy or after surgery, both of which are not conducive to large-scale clinical application nor are suitable for screening high-risk groups. So, in the present study we used serum samples which are relatively easy to obtain. Serum tumor marker detection is a non-invasive detection method for the diagnosis and screening of GC.^16^ In the present case-control study, we profiled the expression levels of 10 MMPs in serum samples from 66 GC and 34 NGD patients using antibody microarrays and found a panel of 5 MMPs (including MMP-3, -8, and -9 and TIMP-1 and -2) which could effectively identify GC and EGC with an AUC of 0.821 (95% *CI*: 0.733-0.909) and 0.749 (95% *CI*: 0.600-0.898), respectively.

There are many methods to analyze the expression levels and activity of MMPs such as enzyme linked immunosorbant assay (ELISA), electrochemiluminescence (ECL), immunohistochemical technique, in situ hybridization, and gelatin zymography. However, all of these methods have their own limitations. Antibody microarray-based technology applied in the present study combines the specificity of ELISA and sensitivity of ECL with a high throughput array.^17^ A study by Choudhry et al. found six proteinases (MMP-1, -2, -8, -10, -12, and -13) with significant differential expression in the serum of the oral squamous cell carcinoma (OSCC) patients and healthy controls.^18^ Among them, the MMP-12 had the highest AUC of 0.836 (95% *CI*: 0.733-0.911) with a sensitivity and specificity of 80.0% and 78.9%, respectively.

### Limitations of the study

First, this study was single-center with a limited sample size. Second, in this study we did not use different methods to verify the differential expression of MMPs and TIMPs. Therefore, future multi-center studies with larger sample sizes and the use of different methods for the analysis of MMP and TIMP expression are required to validate the findings of this study.

## CONCLUSION

The present study unveiled a unique biomarker panel of serum MMP-3, -8, and -9 and TIMP-1 and -2 by measuring serum MMPs and TIMPs, which can be useful in early diagnosis, treatment, and prognostication of patients with GC.

### Authors’ Contribution

**JL & BY:** Study design, data collection and analysis.

**JL:** Manuscript preparation, drafting and revising.

**LYZ & SRL:** Review and final approval of manuscript.

**LYZ:** Take the responsibility and is accountable for the accuracy or integrity of the work.

All authors have seen and approved the final manuscript.
